# The spatial spillover effect of higher SO_2_ emission tax rates on PM_2.5_ concentration in China

**DOI:** 10.1038/s41598-023-31663-z

**Published:** 2023-03-27

**Authors:** Kaifeng Wang, Yu Liu, Shaochen Wang, Chengpeng Li

**Affiliations:** 1 Institute for Environment and Development, Guangdong Academy of Social Sciences, Guangzhou, China; 2 School of Resource and Environmental Sciences, Wuhan University, Wuhan, China

**Keywords:** Environmental sciences, Environmental social sciences

## Abstract

In this paper, the adoption of SO_2_ emission tax rates higher than the legal minimum standard is regarded as a noteworthy policy reform in China (quasi-natural experiment), and a spatial Difference-in-Differences (Spatial-DID) model is constructed to test the direct effects (local effects) and indirect effects (spatial spillover effects) of SO_2_ emission tax policy reform on PM_2.5_ concentrations in the atmosphere of 285 China’s cities. The estimation and calculation results of the Spatial-DID model show that the SO_2_ emission tax policy reform can significantly reduce local PM_2.5_ concentration and significantly promote PM_2.5_ concentration in surrounding areas. The results of heterogeneity analysis show that the SO_2_ emission tax policy reform can produce a relatively more beneficial spatial spillover effect in eastern cities and higher administrative level cities, while the pollutants emission rights trading and the reform of NO_x_ emission tax rates can produce beneficial spatial spillover effects when cooperating with the reform of SO_2_ emission tax rates. The results of the mediation effect analysis show that the higher SO_2_ emission tax rate can aggravate the surrounding PM_2.5_ pollution by promoting the aggregation level of industrial production factors and the industrial SO_2_ emission intensity in the surrounding areas, which can support the existence of the pollution heaven effect.

## Introduction

Although the PM_2.5_ in the atmosphere of China has declined steadily and significantly in recent years, but the average atmospheric concentration of PM_2.5_ in China’s cities (30 μg/m^3^, in 2021) is still five times higher than the latest World Health Organization's standard (5 μg/m^3^), and the days with PM_2.5_ as the primary pollutant accounts for the highest proportion of the total days with air quality lower than standard (39.7%, see https://www.mee.gov.cn/hjzl/sthjzk/zghjzkgb). In addition, the *Global Air Quality Report 2021* released by IQAir also shows that the 15 cities with the highest atmospheric PM_2.5_ concentrations in East Asia are all located in China (https://www.iqair.com/us/blog/press-releases/WAQR_2021_PR). The above data all suggest that PM_2.5_ remains one of the biggest threats to urban air quality in China.

In order to effectively alleviate the air pollution issues represented by PM_2.5_, China has continuously strengthened the tax system on the emission of PM_2.5_ precursors such as SO_2_, NO_x_,^1^ etc. In mainland China before 2018, the pollutants discharge fee system effectively played the role of an environmental protection tax system^2,3^ and formally transformed into an environmental protection tax system after 2018^4^.

The tax on SO_2_ emissions has always been one of the main components of environmental protection taxes (discharge fees). Since 2007, China's environmental protection tax system (in this paper, both the discharge fee and environmental protection tax paid for SO_2_ emissions are collectively referred to as SO_2_ emission tax to avoid confusion) has undergone several rounds of reform, and the type of environmental protection tax that has experienced the earliest, most frequent, and largest reform is just the SO_2_ emission tax^5^, of which primary reform orientation is to promote the tax rate. However, an issue still worth exploring and testing is whether a relatively higher SO_2_ emission tax rate can simultaneously reduce PM_2.5_ pollution in both local areas and adjuncts. This is also the core issue focused on by this paper.

In academia, some studies have tested the pollution reduction effect of the environmental protection tax system from different perspectives. Blackman found that the sewage discharge fees in Colombia effectively reduced pollution^6^, and Wang et al. find that the reform from sewage charge to environmental protection tax effectively reduced pollutant emissions^7^. Guo et al.^5^ use the quasi-natural experimental method to test and confirm that China's pilot sewage charge reform in 2007 significantly reduced the emission intensity of SO_2_.

Many previous studies regard PM_2.5_ as the key policy objective or policy evaluation scale of environmental protection tax (sewage charge), and pay attention to the relationship between PM_2.5_ and policy strength. For example, Ye and An find that the carbon tax significantly improves the air quality in countries with high concentrations of PM_2.5_ in their quasi-natural experiment^8^; Xu et al.^9^ and Hu et al.^10^ both find through mathematical model analysis that higher tax rates can simultaneously reduce greenhouse gas and PM_2.5_ emissions; Rith et al.^11^ find that imposing higher tax rates on gasoline and vehicles would help reduce pollutant emissions and public health risks in Manila. Han and Li^12^ find that the environmental protection tax rate and tax revenue for air pollutants significantly affected the concentration of PM_2.5_ in China; Chien et al.^13^ find that environmental taxes in the United States have played an important role in reducing haze pollution such as PM_2.5_.

As an improvement of the above studies, this paper establishes a quasi-natural experimental framework based on the spatial Difference-in-Differences (Spatial-DID) model and tests the direct effect (local effect) and indirect effect (spatial spillover effect) of SO_2_ emission tax rate reform on PM_2.5_. Compared with previous studies, the main marginal contributions of this paper are:

First, although many previous studies have regarded PM_2.5_ as the goal and evaluation criterion of the environmental protection tax (discharge fee) policy^7,8,13^, these studies usually pay less attention to the spatial spillover effect of PM_2.5_. Using panel data from 285 China's cities (2004–2019), this paper can conduct a more detailed test of the spatial spillover effect of SO_2_ emission tax policy. The deficiencies found in the above tests can also provide essential inspiration for future improvement of relevant policies and systems.

**Figure 1:**
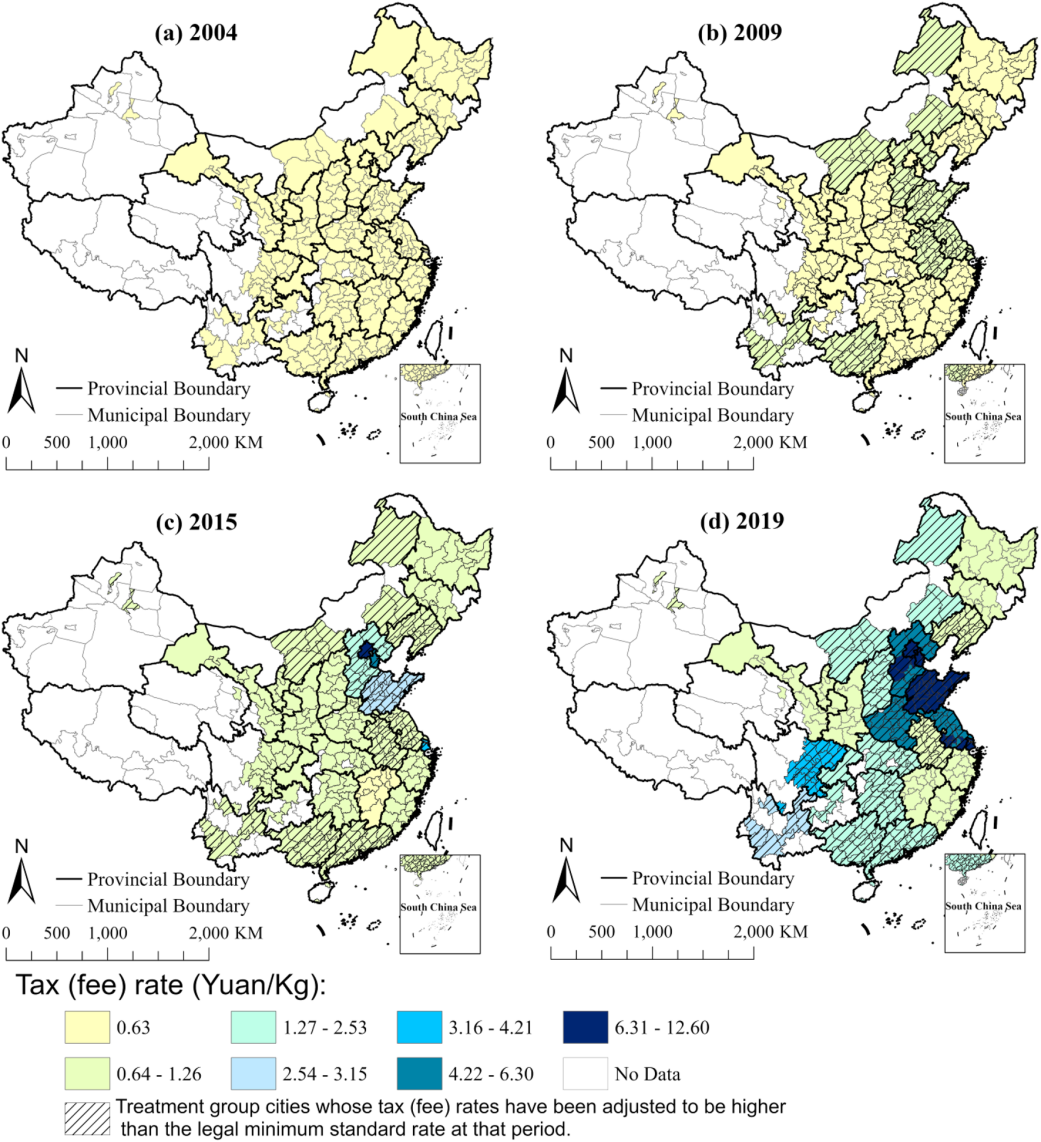
Adjustment of SO_2_ emission tax rate in China. Note: The basic map resources come from Institute of Geographic Sciences and Natural Resources Research (IGSNRR).

Second, the design of Difference-in-Differences (DID) in this paper is to regard the adoption of SO_2_ emission tax rates that are higher than the legal minimum standard (also higher than the other cities which always follow the newest legal minimum tax rate, see Fig. 1) in part of cities as a policy reform worthy of focusing on. Compared with most DID studies in the same field^4,5,8,14^, the above design can help accurately identify the historical changes and regional differences in the tax rate.

Next, the structure of the rest of this paper is as follows: section “Mechanisms and hypotheses” analyzes the theoretical mechanism and puts forward the hypothesis, section “Methodology and data” introduces the methodology and data, section “Results and discussions” reports and discusses the results, and section “Conclusions” is the conclusions.

## Mechanisms and hypotheses

According to the pollution heaven hypothesis, a higher SO_2_ emission tax rate is also possible to force some highly polluting production activities to relocate (through the relocation of production departments, outsourcing, etc.) and lead to increased pollution in surrounding areas^15,16^. Of course, the above spatial spillover effect and its mechanism are still worth testing, so the main hypotheses to be tested in this paper are:

H1: Enabling SO_2_ emission tax rates higher than the national legal minimum standard (so it is also higher than other regions that always follow the minimum standard) has a promotion effect on the atmospheric PM_2.5_ concentration in the surrounding cities (i.e. spatial spillover effect is positive).

Due to the cross-regional migration of high pollution industrial firms^16^ caused by SO_2_ emission tax rates, this paper will also test two sub-hypotheses to explain the mechanism of the spatial spillover effect (if hypothesis H1 can be confirmed):

H1-a: Enabling SO_2_ emission tax rates higher than the national legal minimum standard can increase the atmospheric PM_2.5_ concentration of the surrounding cities by increasing their industrial SO_2_ emission intensity.

H1-b: Enabling SO_2_ emission tax rates higher than the national legal minimum standard can increase the atmospheric PM_2.5_ concentration of the surrounding cities by promoting their accumulation of industrial production factors.

## Methodology and data

### Empirical research design

#### Spatial-DID model

This paper establishes a Spatial-DID model based on the spatial Durbin model (SDM), and then judges whether it should degenerate into a spatial lag model (SLM) or a spatial error model (SEM)^17,18^ according to the test methods provided by Elhorst^19^. The expression of the base-line Spatial-DID model in this paper is:

1 $$\begin{aligned} PM_{i,t} & = \rho_{0} \sum\limits_{j = 1}^{n} {W_{ij} PM_{j,t} } + \alpha_{1} POST_{i,t} \times FSDIOX_{i,t} + \rho_{1} \sum\limits_{j = 1}^{n} {W_{ij} (POST_{j,t} \times FSDIOX_{j,t} )} \\ & \;\;\quad + X_{i,t}^{{}} \beta + \sum\limits_{j = 1}^{n} {W_{ij} X_{j,t} } \theta + f_{i} + \mu_{t} + \varepsilon_{i,t} \\ \end{aligned}$$

In Eq. (1), the interpreted variables *PM *_*i,t*_ are defined as the atmospheric PM_2.5_ concentration of the sample city. $\sum\limits_{j = 1}^{n} {W_{ij} PM_{j,t} }$ is the spatial lagged term of interpreted variable. The letter *t* in the subscript represents the period (year), while the letters *i* and* j* are the sequence numbers used to distinguish different cities. The letter *n* represents the total number of sample cities, and *W*_*ij*_ is the element in i-th row and j-th column of the row standardized spatial weight matrix *W*, and *W* before row standardize is defined as:

2 $$\begin{aligned} W_{ij}^{{}} & = \left\{ \begin{gathered} {1 \mathord{\left/ {\vphantom {1 {d_{ij}^{{}} ,i \ne j}}} \right. \kern-0pt} {d_{ij}^{{}} ,i \ne j}} \hfill \\ 0,i = j \hfill \\ \end{gathered} \right. \\ i & = 1,2 \ldots ,n \\ j & = 1,2 \ldots ,n \\ \end{aligned}$$

*FSDIOX*_*i,t*_ in Eq. (1) is defined as the SO_2_ emission tax rate, and *POST*_*i,t*_ is a dummy variable for the adjustment of SO_2_ emission tax rate (0 before adjustment and 1 after adjustment, which is specifically defined in section “Data for main explanatory variable (DID variable)” below). The product of the above two (*FSDIOX*_*i,t*_ × *POST*_*i,t*_) is the DID term^20^ of Spatial-DID model. $\sum\limits_{j = 1}^{n} {W_{ij} (POST_{j,t} \times FSDIOX_{j,t} )}$ is the spatial lagged term of DID variable. The terms *f*_*i*_ and *μ*_*t*_ stands for individual fixed effect and time fixed effect respectively. *ε*_*i,t*_ is an independent identically distributed random disturbance term. The k-dimensional row vector x contains all control variables. $\sum\limits_{j = 1}^{n} {W_{ij} X_{j,t} }$ contains the spatial lagged terms of all control variables.

In this paper, the scripts provided by Elhorst^19^ are used to estimate Eq. (1). In order to overcome endogenous and bias, the bias corrected quasi maximum likelihood tester (BC-QMLE) provided by Lee and Yu^21^ is selected as the estimation method of Eq. (1).

Based on the estimated parameters, this paper calculates the impact of DID variable on local PM_2.5_ concentrations (direct effect *c*) and the spatial spillover effect on PM_2.5_ concentrations in surrounding areas (indirect effect *c*^*w*^). In the following, this paper will fully judge the policy effects base on the above two types of effects^19,22^.

### Data and variable selection

#### Data for interpreted variable (PM)

The interpreted variable of the spatial-DID model is the annual average PM_2.5_ concentration (*PM*), its data source is the raster data of global PM_2.5_ concentration estimated by ACAG (Atmospheric Composition Analysis Group) using satellite observation data (see Fig. A.1 in the appendices^23^).

#### Data for main explanatory variable (DID variable)

The main explanatory variable of the Spatial-DID model in this paper is the DID term *POST* × *FSDIOX* in Eq. (1), in which the policy meaning of dummy variable *POST* is a major innovation of this paper. Since 2007, according to the order of the State Council of China, some pilot cities began to gradually adjust the tax rate on SO_2_ emissions from 0.63 Yuan/Kg since 2004 to higher levels (ultimately no less than 1.26 Yuan/Kg). In September 2014, China further extended the legal standard of SO_2_ emission tax rate of no less than 1.26 Yuan/Kg to all regions. Then, on January 1st, 2018, China began to change the pollutants discharge fee system into environmental protection tax system obeying the principle of "tax shifting"^4^. After 2018, at least 19 provinces in China Mainland directly determined their environmental protection tax rates according to their previous discharge fee rates (see Fig. 1).

It is noteworthy that after several reforms, only part of the cities have adopted the tax rate higher than the legal minimum standard, and the rest of the cities just follow the legal minimum tax rate standard at each period (see Fig. 1). Therefore, if the first tax rate adjustment in each city is simply regarded as a quasi-natural experiment^4,5,8,14^, it may cause the treatment group and the control group to be indistinguishable, and most historical changes and regional heterogeneity of SO_2_ emission tax rate will be ignored.

Therefore, in this paper, *POST* = 1 means the tax rates of SO2 emissions in the sample cities (206 cities in total) have been adjusted to be higher than the legal minimum standard (*POST* = 0 when this reform has not been launched). Other cities that always follow the statutory minimum tax rate are included in the control group (*POST ≡*0).

Moreover, this paper further combines the continuous value of the actual tax rate to construct a DID variable *POST* × *FSDIOX*^20,24^, in which *FSDIOX* is defined as the real tax rate on SO_2_ emissions (Yuan/Kg, which is deflated by using the price index of the province where the city is located).

Of course, considering the lagging nature of the policy effects, the above-mentioned DID variable has been adjusted to its 1-period lagged term.

#### Data for control variables

The first category of control variables is the population density (*POPUD*), economic development (real GDP per capita, *GDPPC*), and technological progress (R&D personnel as a percentage of total urban employment, *RDEMP*) selected with reference to the STIRPAT model (Stochastic impacts by Regression on Population, Affluence, and Technology)^25^. Since economic development may have nonlinear effects^26^, the squared term of the variable GDPPC is also included in Eq. (1).

The second category is the factors related to infrastructure and energy. The specific indicators used in this paper are per capita urban road area (*ROAD*, to reflect the improvement of urban infrastructure) and gas popularization rate (*GASR*, to simultaneously reflect the popularity of clean energy and the perfection of energy infrastructure).

The third category is the natural environment and climate factors. Specifically: The Normalized Difference Vegetation Index (*NDVI*), which is indicated by the data from the Institute of Geographic Sciences and Natural Resources Research (IGSNRR, http://www.resdc.cn/), Chinese Academy of Sciences (CAS). Temperature (*TEMP*) is indicated by the data from the MERRA-2 raster dataset of the Global Modeling and Assimilation Office (GMAO)^27^. Besides, the data on atmospheric pressure (*PRSD*), wind speed (*WIN*), and air humidity (*RHU*) are extracted from the Dataset of Daily Climate Data from Chinese Surface Stations, provided by the National Meteorological Information Center of China (http://data.cma.cn).

The data of the variables *GDPPC*, *POPUD*, *RDEMP*, *ROAD*, and *GASR* comes from *The Yearbook of China's Cities* or *The Yearbook of China's Urban Construction*. These yearbooks directly provide the above data at the prefecture-level city level, of which the data of 285 prefecture or higher-level cities are extracted into the panel data of this paper (the total number of the prefecture or higher-level cities in China is 337, of which 52 cities are not included in the sample cities of this paper since their series data missing problems).

The original data of variables *PM*, *NDVI*, and *TEMP* are geographic rasters. The original data of *PRSD*, *WIN*, and *RHU* are the data of monitoring stations, and this paper uses the Kriging interpolation method to transform them into raster form. Subsequently, this paper calculates the annual mean of rasters within the boundary of 285 sample cities to convert all above the geographic rasters into panel data.

Finally, this paper unifies the data of the above variables into the panel data of 285 sample cities, and the data period is 2004–2019. The descriptive statistics of all the above data are reported in Table 1.

**Table 1:** Descriptive statistics.

Variable	Indicator	Number of Obs	Mean Value	Std. Dev	Max	Min	Unit	Role and references
*PM*	PM_2.5_ concentration (van Donkelaar et al., 2021)	4560	45.313	15.285	108.634	13.454	μg/m^3^	Interpreted variable (van Donkelaar et al.^23^)
*POST*	Dummy variable for discharge tax reform	4560	0.310	0.462	1.000	0.000	–	Components of the main explanatory variable (i.e., the DID term, Jia et al.^20^)
*FSDIOX*	Real tax rate on SO_2_ emission	4560	0.735	0.717	7.646	0.188	Yuan / Kg	
*GDPPC*	Per capita real GDP	4560	16,970.941	12,543.812	92,522.068	1283.188	Yuan/people	Control the impact of social and economic development levels on air pollution (Dietz and Rosa^25^)
*POPUD*	Population density	4560	3661.339	2822.769	27,203.060	27.000	People/Km^2^	
*RDEMP*	Proportion of R & D employees in population	4560	0.178	0.236	2.784	0.009	%	
*ROAD*	Road area per capita	4560	36,873.451	33,569.976	359,608.498	376.770	m^2^/people	Control the impact of the infrastructure of transportation and energy on air pollution (Liu et al.^28^; Zhang and Wang^29^)
*GASR*	Gas penetration rate	4560	22.411	18.199	100.000	0.483	%	
*NDVI*	Vegetation index	4560	72.595	13.450	89.545	9.689	%	Control the inhibition and absorption of vegetation on PM_2.5_ (Liu et al.^28^)
*PRSD*	Annual mean atmospheric pressure	4560	957.914	62.922	1016.810	693.283	Hpa	Control the impact of meteorological factors on PM2.5 pollution (Liu et al.^30^; Liu et al.^28^; Yan et al.^16^)
*TEMP*	Annual mean temperature (absolute temperature)	4560	286.984	5.182	298.902	271.941	Degree	
*WIN*	Annual mean wind speed	4560	2.129	0.416	3.658	1.047	m/s	
*RHU*	Annual mean relative humidity	4560	65.650	9.944	80.083	32.119	%	

## Results and discussions

### Empirical results

#### Parallel trend test

Referring to Beck^31^, the parallel trend test model of Spatial-DID model is defined as:

3 $$\begin{aligned} PM_{i,t} & = \rho_{0} \sum\limits_{j = 1}^{N} {W_{ij} PM_{j,t} } + \sum\limits_{{k = 1 - T_{b} }}^{5} {\alpha_{k} T_{i,t}^{ - k} } + \sum\limits_{{s = 1 + T_{b} }}^{10} {\alpha_{s} T_{i,t}^{ + s} } + \sum\limits_{{k = 1 - T_{b} }}^{5} {\rho_{k} \sum\limits_{j = 1}^{N} {W_{ij} T_{j,t}^{ - k} } } + \sum\limits_{{s = 1 + T_{b} }}^{l} {\rho_{s} \sum\limits_{j = 1}^{10} {W_{ij} T_{j,t}^{ + s} } } \\ & \;\;\; + X_{i,t}^{{}} \beta + \sum\limits_{j = 1}^{N} {W_{ij} X_{j,t} } \theta + f_{i} + \mu_{t} + \varepsilon_{i,t} \\ \end{aligned}$$

In Eq. (3), if the launch time of policy reform in the city *i* is 2009, and there is *t* = 2009-*k* (*k* ∈ *Z*^+^ and *k* ≤ *p*) or *t* = 2009 + *s* (*s* ∈ *Z*^+^ and *s* ≤ *l*), then $T_{i,t}^{ - k}$ (or $T_{i,t}^{ + s}$) equals 1, otherwise $T_{i,t}^{ - k}$ (or $T_{i,t}^{ + s}$) equals 0. According to the time span of panel data, this paper sets the maximum values of *k* and *s* as 5 and 10 respectively, and selects the first period (− 1 period) before the policy launch as the base period (*T*_*b*_) of the parallel trend test.

**Figure 2:**
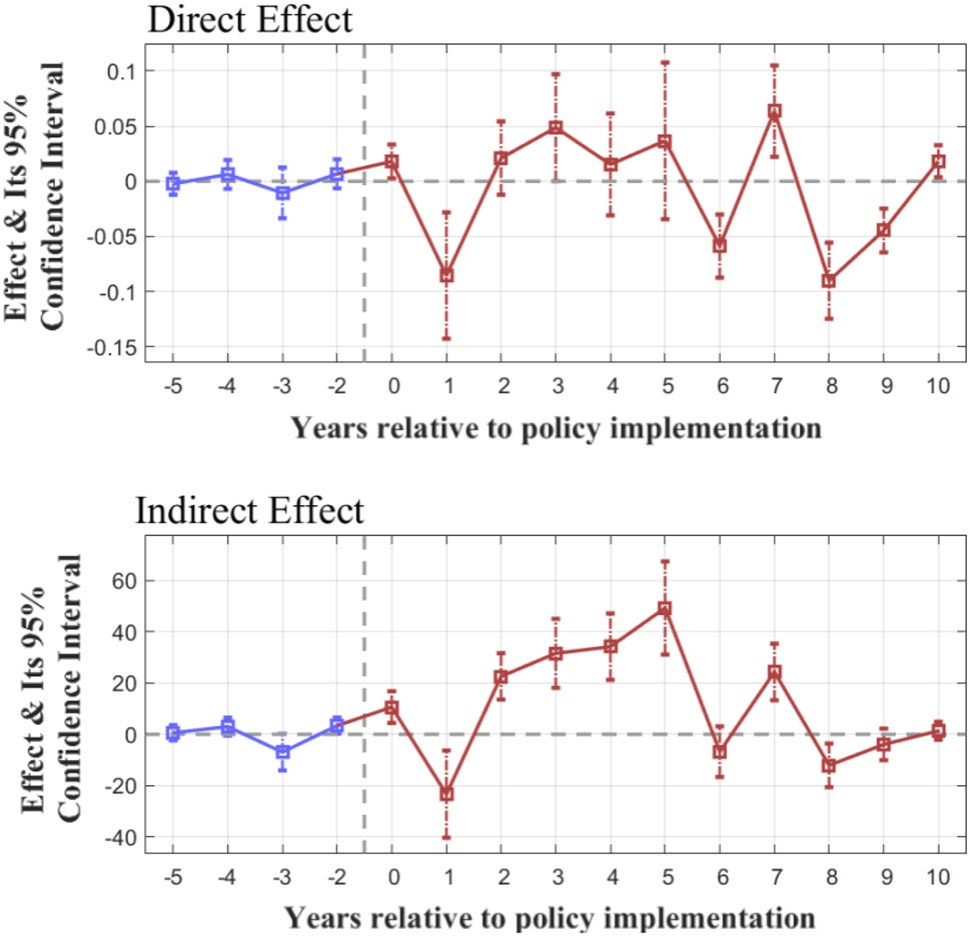
Parallel trend test results.

Referring to Jia et al.^20^, the test results of the parallel trend hypothesis are indicated by both the direct and indirect effects (95% confidence intervals were also reported, see Fig. 2). It can be seen that before the base period (the period before the policy reform^31^), the direct and indirect effects of $T_{i,t}^{ - k}$ never significantly deviate from 0, indicating that the policy has not taken effect; however, after the base period, the two types of effects of $T_{i,t}^{ + s}$ begin to deviate significantly 0, in which the direct effect is a mainly negative effect, and the indirect effect is a mainly positive effect. The above results prove that the parallel trend hypothesis is confirmed.

#### Selective test of spatial econometric models

The implementation of spatial econometric analysis must be based on the existence of spatial autocorrelation of the explained variables. The results of the spatial autocorrelation tests based on global Moran's I test and Moran Scatter (reported in Appendix A2) show that PM_2.5_ concentration has significant positive spatial autocorrelation (p-value < 0.05). Therefore, to control the spatial spillover effect of PM_2.5_ (cross-boundary transmission of pollution), it is necessary to select the spatial econometric model as the empirical test tool.

The empirical results of this base-line model (Model A) are reported in Table 2. The spatial lag coefficient of the interpreted variable (PM_2.5_ concentration) is significantly positive (0.722, p-value < 0.01), which also confirms the significant positive spatial autocorrelation of PM_2.5_ concentration, meaning that Model A has identified and extracted the cross-boundary impact of surrounding areas on local PM_2.5_ pollution.

**Table 2:** Estimation results.

Variables		Robustness tests					Heterogeneity analysis				
	Model A	Model B	Model C	Model D	Model E	Model F	Model G	Model H	Model I	Model J	Model K
	Base-line Model	PSM-Spatial-DID	Spatial-DID-2SLS Model	Spatial weight matrix: *We*	Interpreted variable: *PMCH*	Interpreted variable: *PME*	Eastern cities (*EAST*)	Administrative level (*CLEVEL*)	Pilot cities of emission rights trading (*PET*)	Key regions of APPCAP (*PCAP*)	NO_x_ emission tax rate reform (*REFNF*)
*POST* × *FSDIOX*	− 0.039***	− 0.047***	− 0.02**	− 0.024***	− 0.033***	− 0.117***	− 0.049***	− 0.02**	− 0.032***	− 0.025***	− 0.034***
	(− 6.451)	(− 8.33)	(− 2.33)	(-4.786)	(− 7.716)	(− 8.266)	(− 4.661)	(− 2.09)	(− 4.294)	(− 3.453)	(− 3.563)
*W* × *POST* × *FSDIOX*	0.119***	0.148***	0.022	0.216***	0.106***	0.290***	0.184***	0.34***	0.15***	0.110***	0.144***
	(4.225)	(5.648)	(0.64)	(3.063)	(5.211)	(4.504)	(3.295)	(3.164)	(3.594)	(2.933)	(3.104)
*W* × *PM* (Interpreted Variable)	0.981***	0.977***	2.09***	− 0.075	–	–	0.981***	0.984***	0.983***	0.983***	0.981***
	(304.057)	(244.672)	(19.54)	(–0.797)	–	–	(304.331)	(361.466)	(340.950)	(339.768)	(303.862)
*W* × *PMCH* (Interpreted Variable)	–	–	–	–	0.986***	–	–	–	–	–	–
	–	–	–	–	(411.608)	–	–	–	–	–	–
*W* × *PME* (Interpreted Variable)	–	–	–	–	–	0.928***	–	–	–	–	–
	–	–	–	–	–	(79.313)	–	–	–	–	–
Control Variables	Controlled	Controlled	Controlled	Controlled	Controlled	Controlled	Controlled	Controlled	Controlled	Controlled	Controlled
											–
*D.E._POST* × *FSDIOX*	− 0.024***	− 0.030***	− 0.019**	− 0.024***	− 0.014***	− 0.109***	− 0.022**	0.062**	− 0.005	− 0.010**	− 0.016**
	(− 4.024)	(− 6.091)	(− 2.257)	(− 4.882)	(− 2.717)	(− 9.713)	(− 1.990)	(2.387)	(− 0.629)	(− 2.393)	(− 2.133)
*I.E._ POST* × *FSDIOX*	4.505***	4.449***	0.029**	0.204***	5.411***	2.737***	8.496***	22.932***	7.534***	5.044***	5.776***
	(2.899)	(3.731)	(2.093)	(2.917)	(3.092)	(2.912)	(3.292)	(3.238)	(3.399)	(2.756)	(3.297)
*D.E._POST* × *FSDIOX* × *EAST*	-	-	–	–	–	–	0.001	–	–	–	–
	–	–	–	–	–	–	(0.083)	–	–	–	–
*I.E._POST* × *FSDIOX* × *EAST*	–	–	–	–	–	–	− 5.309**	–	–	–	–
	–	–	–	–	–	–	(− 2.053)	–	–	–	–
*D.E._POST* × *FSDIOX* × *CLEVEL*	–	–	–	–	–	–	–	− 0.069***	–	–	–
	–	–	–	–	–	–	–	(− 3.424)	–	–	–
*I.E._POST* × *FSDIOX* × *CLEVEL*	–	–	–	–	–	–	–	− 15.264***	–	–	–
	–	–	–	–	–	–	–	(− 2.735)	–	–	–
*D.E._POST* × *FSDIOX* × *PET*	–	–	–	–	–	–	–	–	− 0.036***	–	–
	–	–	–	–	–	–	–	–	(− 3.102)	–	–
*D.E._POST* × *FSDIOX* × *PET*	–	–	–	–	–	–	–	–	− 0.036***	–	–
	–	–	–	–	–	–	–	–	(− 3.102)	–	–
*I.E._POST* × *FSDIOX* × *PCAP*	–	–	–	–	–	–	–	–	–	− 0.043***	–
	–	–	–	–	–	–	–	–	–	(− 4.142)	–
*I.E._POST* × *FSDIOX* × *PCAP*	–	–	–	–	–	–	–	–	–	− 3.209	–
	–	–	–	–	–	–	–	–	–	(− 0.817)	–
*I.E._POST* × *FSDIOX* × *REFNF*	–	–	–	–	–	–	–	–	–	–	− 0.010
	–	–	–	–	–	–	–	–	–	–	(− 1.397)
*I.E._POST* × *FSDIOX* × *REFNF*	–	–	–	–	–	–	–	–	–	–	− 1.703*
	–	–	–	–	–	–	–	–	–	–	(− 1.851)
	–	–	–	–	–	–	–	–	–		–
Observations	4560	3936	4560	4560	4560	4275	4560	4560	4560	4560	4560
Number of cities	285	246	285	285	285	285	285	285	285	285	285
*R*^*2*^	0.9736	0.9768	0.9736	0.9597	0.9858	0.9854	0.9736	0.9737	0.9736	0.9737	0.9736
Wald spatial lag test	401.585***	193.251***	100.208***	555.096***	215.846***	410.056***	400.756***	401.969***	404.417***	401.822***	394.278***
LR spatial lag test	407.764***	201.067***	407.764***	561.502***	224.662***	417.219***	406.938***	409.178***	411.546***	408.468***	400.686***
Wald spatial error test	451.871***	224.211***	109.747***	558.045***	263.812***	413.476***	451.17***	455.437***	456.579***	454.439***	446.576***
LR spatial error test	455.728***	232.09***	455.728***	559.485***	271.616***	417.575***	454.801***	459.925***	460.666***	459.435***	451.002***
LR spatial fixed effects test	11,108.394***	10,086.787***	10,088.703***	11,108.394***	13,335.203***	14,624.605***	11,105.64***	11,116.038***	11,111.6***	11,126.816***	11,055.903***
LR time fixed effects test	3186.68***	3242.986***	1578.417***	3186.680***	6688.258***	1615.188***	3189.912***	3194.548***	3173.147***	3194.900***	3183.774***

Note: The t-statistics are marked in parentheses. *, **, and *** denote significance at 10%, 5%, and 1% levels, respectively. D.E. before the variable symbol indicates direct effect, and I.E. indicates indirect effect. The complete estimation results are reported in Table A.2 in the appendices.

The statistics of the Wald test and LR test about space error and space lag in Table 2 show the selective test results of the spatial econometric model in this paper. It can be seen that the significance level of all Wald test and LR test statistics reaches 1%, indicating that the null hypothesis that the SDM should generate into the spatial lag model (SLM) or spatial error model (SEM) is all rejected. Therefore, SDM should be selected as the modeling basis of the Spatial-DID model in this paper.

#### Effects of main explanatory variable

From Table 2, it can be seen that the SO_2_ emission tax policy reform can produce a significant negative direct effect on the PM_2.5_ concentration in the local atmosphere (p-value < 0.01), and a significantly positive indirect (spatial spillover) effect (p-value < 0.01) on the atmospheric PM_2.5_ concentration of surrounding areas, which are consistent with the parallel trend test results (Fig. 2). Therefore, hypothesis H1 is confirmed.

### Placebo test

**Figure 3:**
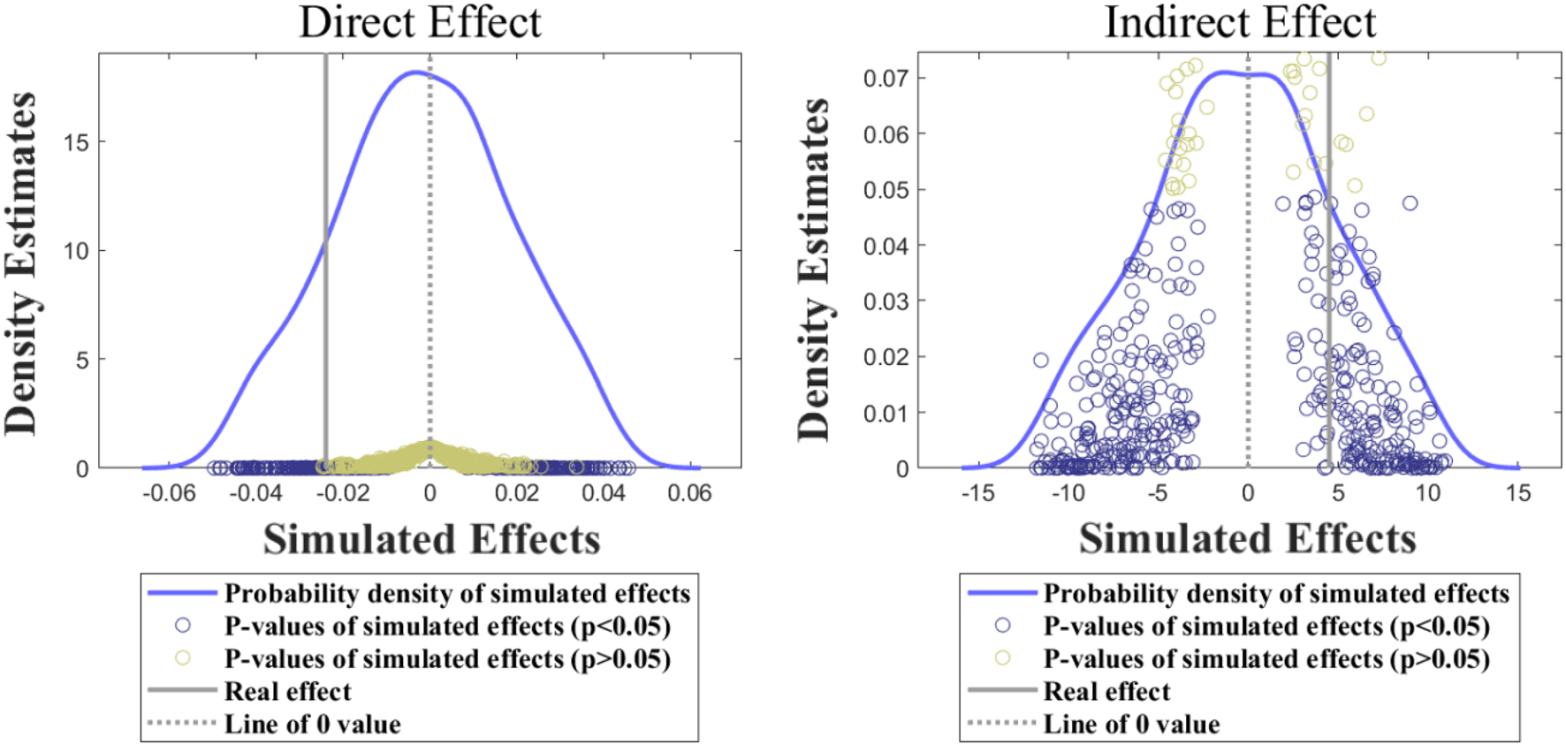
Placebo test results.

The placebo test (to test whether the empirical results are affected by unobserved factors) is to randomly assign false treatment groups and time points of policy reform in a false DID variable of the Spatial-DID model, and then process the estimation and test the effects by using a Spatial-DID model with this false DID variable. This paper repeated the above test 1000 times and obtained the distribution of direct and indirect effects (see Fig. 3). It can be seen from Fig. 3 that, significantly different from the effect of the real DID variable (grey vertical line), the direct and indirect effects of the 1000 placebo tests are concentrated around the 0 value, and the effect is the weakest near the 0 value. This shows that the empirical results of this paper are not caused by unobserved factors^20^, but truly identify and measure the spatial spillover effects brought about by policy reform.

### Robustness tests

In addition to the base-line model (Model A), this paper also established a total of 10 auxiliary models, of which Model B—Model F are robustness test models and Model G—Model K are heterogeneity analysis models. The estimation results and discussion of the above models are described in sections “Robustness tests” and “Heterogneity analysis”.

The purpose of Model B—Model F in this subsection is to judge whether the empirical results of Model A are robust and credible. If the models can still draw empirical conclusions that are generally consistent with Model A after some partial modifications, it indicates that the empirical results of model A are robust. In addition, in a series of robustness tests, the use of propensity score matching (Model B) and instrumental variable (Model C) can also be used to determine whether the empirical conclusions are affected by selective bias and endogenous respectively, thus further proving the effectiveness of Model A.

#### Propensity score matching

In order to avoid selection bias caused by the fact that the treatment group samples may not satisfy the assumption of random selection^20,32^, this paper performs propensity score matching (PSM) on the panel data year by year based on all control variables (reported in Table A.4 of the appendices). The Spatial-DID model using matched data is called the PSM-Spatial-DID model (Model B), and the number of sample cities drops from 285 to 246 in Model B. From Table 2, it can be seen that the direction (sign) of the marginal effect (P-values < 0.01) of the DID variable in Model B is still consistent with the base-line model (Model A), indicating that the empirical results in this paper are not significantly affected by the problem of selective bias.

#### Instrumental variable approach

Referring to Shehata^33^ and Vega and Elhorst^34^, this paper treats the spatial lagged interpreted variable (*W* × *PM*), DID variable (*POST* × *FSDIOX*), and its spatial lagged term as endogenous variables, using instrumental variables (IV) and two-stage Least Squares (2SLS) to overcome endogeneity, thus extending the base-line Spatial-DID model to Spatial-DID-2SLS model (Model C). The IV of the DID variable is the sewage treatment capacity of sample cities (*WDCAPA*). The reason for choosing this IV is that cities with larger sewage treatment capacity usually tend to make up for the huge investment in pollution control by increasing the tax (fee) rate on SO_2_ emission. In addition, refer to Shehata^33^, the 2–4th order spatial lagged terms of the DID variable are selected as IVs of spatial lagged interpreted variable; refer to Vega and Elhorst^34^ and Shao et al.^35^, choose the 1–3th order spatial lag terms of the main IV (*WDCAPA*) are used as IVs of the spatial lagged DID variable.

It can be seen from Table 2 and Table A.5 (in the appendices) that the direct and indirect effects of Model C are not substantially different from the base-line model (Model A), indicating that endogeneity cannot significantly affect the effectiveness of the Spatial-DID model.

#### Replace the spatial weight matrix

An alternative spatial weight matrix *i*s *We* = *W* × *E*, where matrix *E* is defined as:

4 $$\left\{ \begin{gathered} E_{ij}^{{}} { = }\frac{1}{{GDPPC_{i} - GDPPC_{j} }},_{{}} i \ne j \hfill \\ E_{ij}^{{}} { = }0,_{{}} i = j \hfill \\ \end{gathered} \right.$$

In the above formula, *GDPPC* is the average real GDP per capita of sample cities from 2004 to 2019. The estimated results of the Spatial-DID model using *We* as spatial weight matrix (Model D) are reported in Table 2. It can be seen that there are only limited differences between the parameter estimates and effects of Model A and Model D, which reflects the robustness of the empirical results.

#### Replace the interpreted variable

With the comparison between the models after replacing the interpreted variable and the base-line model, this paper further tests the robustness and credibility of the empirical conclusions. There are two alternative interpreted variables used in this paper. The first is PM2.5 atmospheric concentration (*PMCH*) from the China High Air Pollutants (CHAP) dataset^36^, measured in μg/m^3^, its original data is from the simulations based on China's ground monitoring data and satellite remote sensing images using artificial intelligence methods. The second alternative interpreted variable is the PM_2.5_ emission flux (*PME*), which can directly reflect the density of PM_2.5_ (Kg/m^2^) generated and discharged into the atmosphere by each sample city. Its data source is the dataset of Hemispheric Transport of Air Pollution (HTAP). The models using the above two interpreted variables are Model E and Model F. From Table 2, it can be seen that the empirical results of Model E and Model F are not fundamentally different from Model A, which shows that neither changing the source of PM_2.5_ atmospheric concentration data (changing to the data source from China) nor only considering the local emission of PM_2.5_ can essentially change the empirical results of this paper.

### Heterogneity analysis

Referring to Yu and Zhang (2021), this paper further adds the interaction term of city heterogeneity variable and DID term to Eq. (1) to separate and identify the impact of regional characteristics or the interference of other policies. The following heterogeneity variables are selected in this paper:

- Eastern cities (*EAST*), a 0–1 dummy variable which distinguishes eastern China from other regions.
- City level (*CLEVEL*), which represents the cities’ administrative levels by the values between 1 and 3.
- Pollutant emission rights trading pilot cities (*PET*), which is a 0–1 dummy variable representing the launch of trading.
- Key regions dummy variable (*PCAP*) of the Air Pollution Prevention and Control Action Plan (APPCAP), represents the launch of the APPCAP in the key regions. Here, the key regions refer to the three major areas of the Beijing-Tianjin-Tangshan region, the Yangtze River Delta, and the Pearl River Delta which are the focus of attention in the APPCAP (http://www.gov.cn/zhengce/content/2013-09/13/content_4561.htm).
- NO_x_ emission tax rate reform (*REFNF*); NO_x_ is also the main precursor of PM_2.5_; during 2004–2019, the NO_x_ emission tax rates are also adjusted in many regions of China. Similar to the DID term in Eq. (1) (*POST* × *FSDIOX*), the definition of *REFNF* is similar to the DID term of SO_2_ emission tax rate reform in Eq. (1) (*POST* × *FSDIOX*), which equals to variable *POSTN* (a 0–1 dummy variable representing the adoption of NO_x_ emission tax rates higher than the national statutory minimum standard) multiplied by the real NO_x_ emission tax rate *FNOX*.

The results of the heterogeneity analysis are reported in Table 2. It can be seen that the direct effect of the interaction term *POST* × *FSDIOX* × *EAST* is not significant, but its indirect effect is significantly negative (P-values < 0.05), indicating that the policy reform can have a relatively weak PM_2.5_ pollution aggravation effect on the surrounding areas of each eastern city, which is similar to the conclusion of Jia et al.^20^. The interaction terms *POST* × *FSDIOX* × *CLEVEL* can simultaneously produce negative direct effects and indirect effects (P-values ≤ 0.05), indicating that the policy reform in higher administrative level cities can produce a larger local PM_2.5_ pollution suppression effect and a weaker surrounding PM_2.5_ pollution aggravation effect, this may be due to the fact that China's higher administrative level cities are usually subject to stricter environmental supervision^1^.

At the same time, the heterogeneity analysis also shows the synergy between different environmental regulation policies. it can be seen from Table2 that the Interactive terms *POST* × *FSDIOX* × *PET* (in Model I) and *POST* × *FSDIOX* × *REFNF* (in Model K) both can produce negative indirect effects (P-values ≤ 0.1), which shows that the pollutants emission rights trading and the NO_x_ emission tax rate reform can bring beneficial spatial spillover effects when cooperating with the reform of SO2 emission tax rates. The interactive term *POST* × *FSDIOX* × *PCAP* (in Model J) also produces a negative but not significant indirect effect, which may be due to the late start (after 2013) and gradual implementation of APPCAP (see http://www.gov.cn/zhengce/content/2013-09/13/content_4561.htm).

Moreover, it can be seen from Table 2 that the interaction with above policies will not fundamentally change the spatial spillover effect of the SO_2_ emission tax rate reform (the indirect effects of *POST* × *FSDIOX* in Table 2).

### Mechanism analysis (mediation effects)

In this paper, the following mediation effect analysis method is used to test the hypotheses (H1-a and H1-b) involving the generation mechanism of the spatial spillover effects:

First, use the following model to estimate the impact of the DID variable on the mediator *M*_*it*_:

5 $$\begin{aligned} M_{i,t}^{{}} & = \rho_{0} \sum\limits_{j = 1}^{n} {W_{ij} M_{j,t} } + \alpha_{1} POST_{j,t} \times FSDIOX_{j,t} + \rho_{1} \sum\limits_{j = 1}^{n} {W_{ij} POST_{j,t} \times FSDIOX_{j,t} } \\ & \;\; + X_{i,t}^{{}} \beta + \sum\limits_{j = 1}^{N} {W_{ij} X_{j,t} } \theta + f_{i} + \mu_{t} + \varepsilon_{i,t} \\ \end{aligned}$$

According to the parameter estimates of Eq. (5), the direct effect (effect* a*) and indirect effect (i.e. spatial spillover effect* a*^*w*^) of the DID variable on the mediator *M*_*it*_ can be calculated. Then, the mediator *M*_*it*_ is added to the following equation:

6 $$\begin{aligned} PM_{i,t} & = \rho_{0} \sum\limits_{j = 1}^{n} {W_{ij} PM_{j,t} } + \alpha_{0} M_{i,t} + \alpha_{1} POST_{i,t} \times FSDIOX_{i,t} + \rho_{2} \sum\limits_{j = 1}^{N} {W_{ij} M_{j,t} } \\ & \;\;\; \quad+ \rho_{1} \sum\limits_{j = 1}^{n} {W_{ij} POST_{j,t} \times FSDIOX_{j,t} } + X_{i,t}^{{}} \beta + \sum\limits_{j = 1}^{N} {W_{ij} X_{j,t} } \theta + f_{i} + \mu_{t} + \varepsilon_{i,t} \\ \end{aligned}$$

According to the parameters of Eq. (6), the direct effect (effect *b*) of the mediator *M*_*it*_ on PM_2.5_ can be calculated^19^. After identifying and stripping the mediation mechanism contained in direct effect (consisting of effects *a* and *b*) and the mediation mechanism contained in indirect effect (consisting of effects *a*^*w*^ and *b*), the actual direct effect (effect *c*') and actual spatial spillover effect (effect *c*^*w*^') can be measured. Based on the above direct effects and indirect effects (rather than parameters^20^), the logic and criterion refer to Baron and Kenny^37^, MacKinnon et al.^38^, etc. are used to analysis the mediation effects.

According to the hypothesis in section “Mechanisms and hypotheses”, this paper uses two categories of mediators: the first is the SO_2_ emission intensity (*SDIOXINT*), which is indicated by the proportion of SO_2_ emissions to industrial outputs, and its time span is 2004–2017. The second category contains two types of industrial production factors: (a) *INDEMP*, which means the proportion of industrial labor in the city to total employment; (b) *INDFA*, which means the real industrial fixed assets per employee.

**Figure 4:**
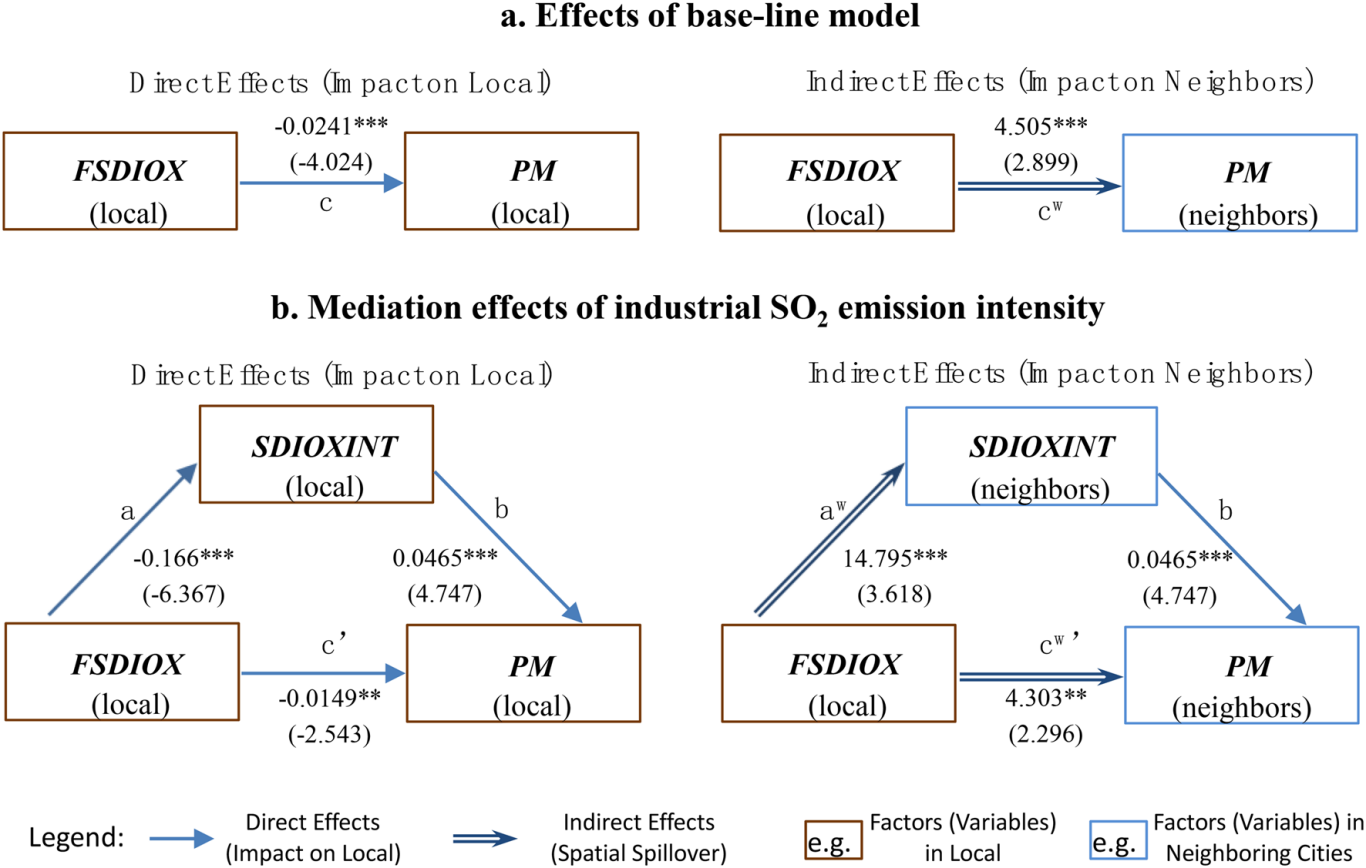
The mediation effects of industrial SO_2_ emission intensity. Notes: (**a**) The t-statistics are marked in parentheses. *, **, and *** denote significance at 10%, 5%, and 1% levels, respectively. (**b**) It should be noted that some mediation effect studies^39^ also refer to effect *c'*′ as a direct effect and refer to effect *a* × *b* as an indirect effect, so readers should pay attention to the differences between the definitions in the above studies and those in this paper.

It can be seen from the small graph on the right side of Fig. 4 that the policy reform can greatly increase the industrial SO_2_ emission intensity of surrounding cities (effect *a*^*w*^, P-value ≤ 0.01), which can further increase the PM_2.5_ pollution in surrounding cities with the help of effect *b*. After separating the above mediation effect, the actual indirect effect *c*^*w*^′ (4.303, P-value ≤ 0.05) of the policy reform on the local PM_2.5_ concentration is smaller than the original effect *c*^*w*^ (4.505) (in Model A), indicating that the policy reform can result in more serious surrounding PM_2.5_ pollution by increasing the intensity of industrial SO_2_ emissions in surrounding cities. Based on the above results, hypothesis H1-a is confirmed.

**Figure 5:**
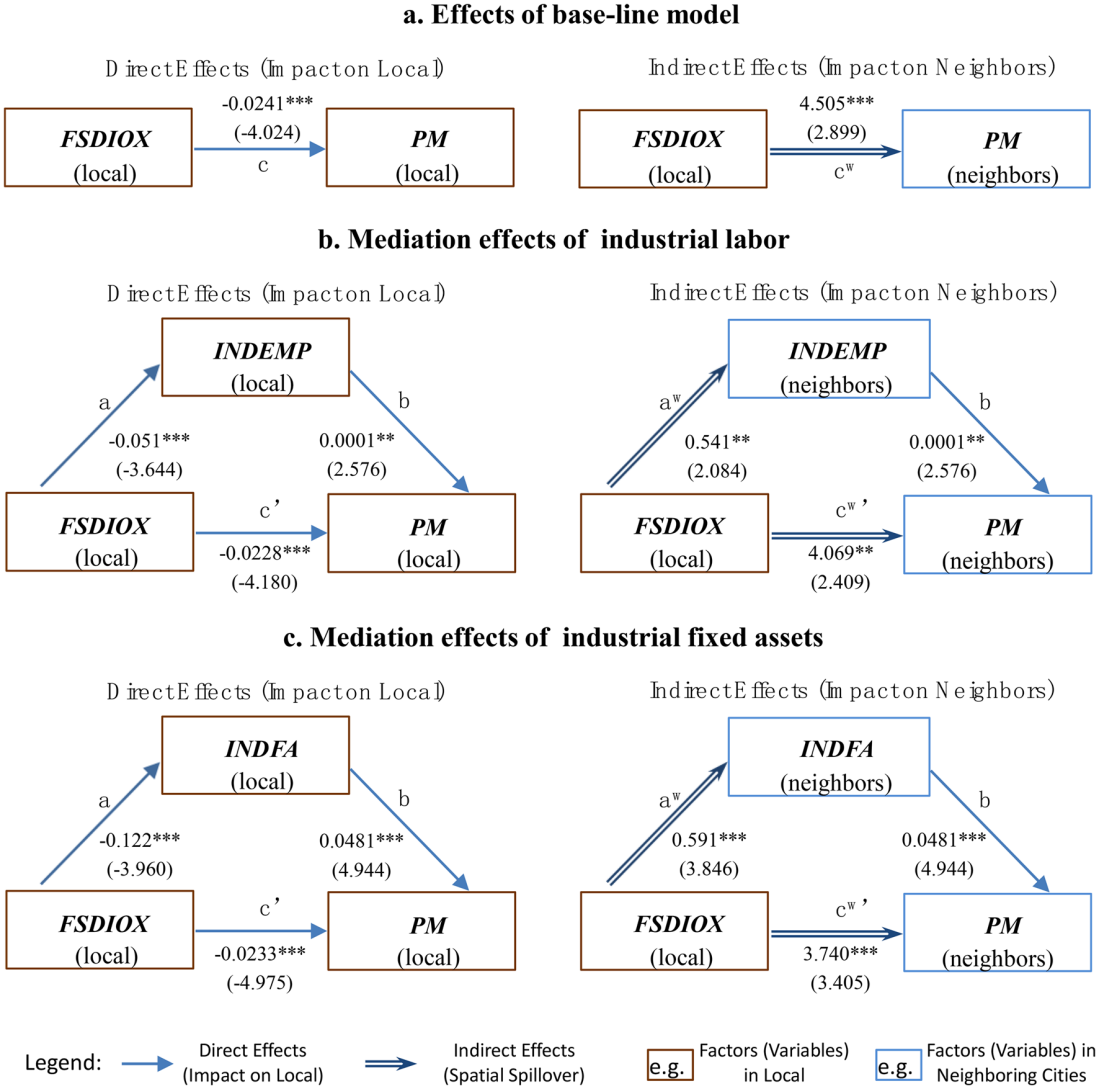
The mediation effects of industrial production factors. Note: The t-statistics are marked in parentheses. *, **, and *** denote significance at 10%, 5%, and 1% levels, respectively.

It can also be seen from Fig. 5 that the SO_2_ emission tax policy reform has an improvement effect (effect *a*^*w*^) on the variables *INDEMP* and *INDFA* in the surrounding cities (P-values ≤ 0.05), and both of them can further increase the concentration of PM_2.5_ in surrounding cities (effect *b*). Further, comparing Fig. 5a–c, it can be seen that the actual indirect effects *c*^*w*^*'* (P-values ≤ 0.05) after separating the above mediation effects are both far smaller than the original effect *c*^*w*^ (6.385) (in Model A), indicating that the policy reform can result in more serious surrounding PM_2.5_ pollution by increasing the concentration of industrial production factors in surrounding cities. Based on the above results, hypothesis H1-b is also confirmed. The above results together provide strong evidence for the existence of the pollution heaven effect in the SO_2_ emission tax policy reform^15,16^.

## Conclusions

After considering the primary results, we conclude the following:

1. The policy reform of SO_2_ emission tax (enabling SO_2_ emission tax rates higher than the legal minimum standard at that time) can significantly reduce the concentration of PM_2.5_ locally, but significantly increase PM_2.5_ concentrations in surrounding areas.
2. The policy reform of SO_2_ emission tax can lead to a relatively weaker effect on the aggravation of PM_2.5_ pollution in the surrounding areas of eastern cities, while the policy reform in high-level cities can lead to weaker surrounding PM_2.5_ pollution aggravating effect. The pollutants emission rights trading and the reform of NO_x_ emission tax rates can produce beneficial spatial spillover effects when cooperating with the reform of SO_2_ emission tax rates.
3. Reform of the SO_2_ emission tax policy can cause the surrounding PM_2.5_ pollution to aggravate by promoting the accumulation of surrounding industrial production factors and the increase in SO_2_ emission intensity.

The policy suggestions according to the above issues are mainly:

1. The most direct countermeasure is to expand the geographical scope of the SO_2_ emission tax rates higher than the legal minimum standard so that more regions can adopt the discharge tax rates higher than the legal minimum standards that more China’s cities can share the beneficial impact of the discharge tax policy. Otherwise, China’s cities should improve the ecological compensation mechanism for enterprises with damaged interests after the burden of taxes is increased, and provide sufficient subsidies or incentives for their green transformation, and the relocation costs of highly polluting enterprises should also be increased through inter-city cooperation.
2. Promote resource flow and sharing more flexibly, such as adopting a more flexible employment mechanism for green technology R&D personnel and emissions trading practitioners, establishing a closer green technology sharing and collaboration relationship, etc. Then, the spillover of resources required for the environmental governance in terms of R&D, operation, and management can offset the negative spatial spillover effect that may be caused by the taxes on pollutant emissions.

A probable inadequacy of this paper is that due to the lack of data availability of prefecture-level cities, this paper cannot use the actual collection strength index such as the total amount of discharge tax collection. The above problem also provides a possible future improvement direction for our future research.

## Supplementary Information

Supplementary Information.

## Data Availability

The datasets used and analysed during the current study available from the corresponding author on reasonable request.
