# Supplementary material for: The spatial spillover effect of higher SO2 emission tax rates on PM2.5 concentration in China
Source: Sci Rep. 2023 Mar 27;13:4966. doi: 10.1038/s41598-023-31663-z (PMC10042812; doi:10.1038/s41598-023-31663-z)
Supplement: Supplementary file 1 — Supplementary Information. [file 41598_2023_31663_MOESM1_ESM.docx]

**Appendices**

**The spatial spillover effect of higher SO2 emission tax rates on PM2.5 concentration in China**

**A1. Example of PM2.5 Atmospheric Concentration Data**


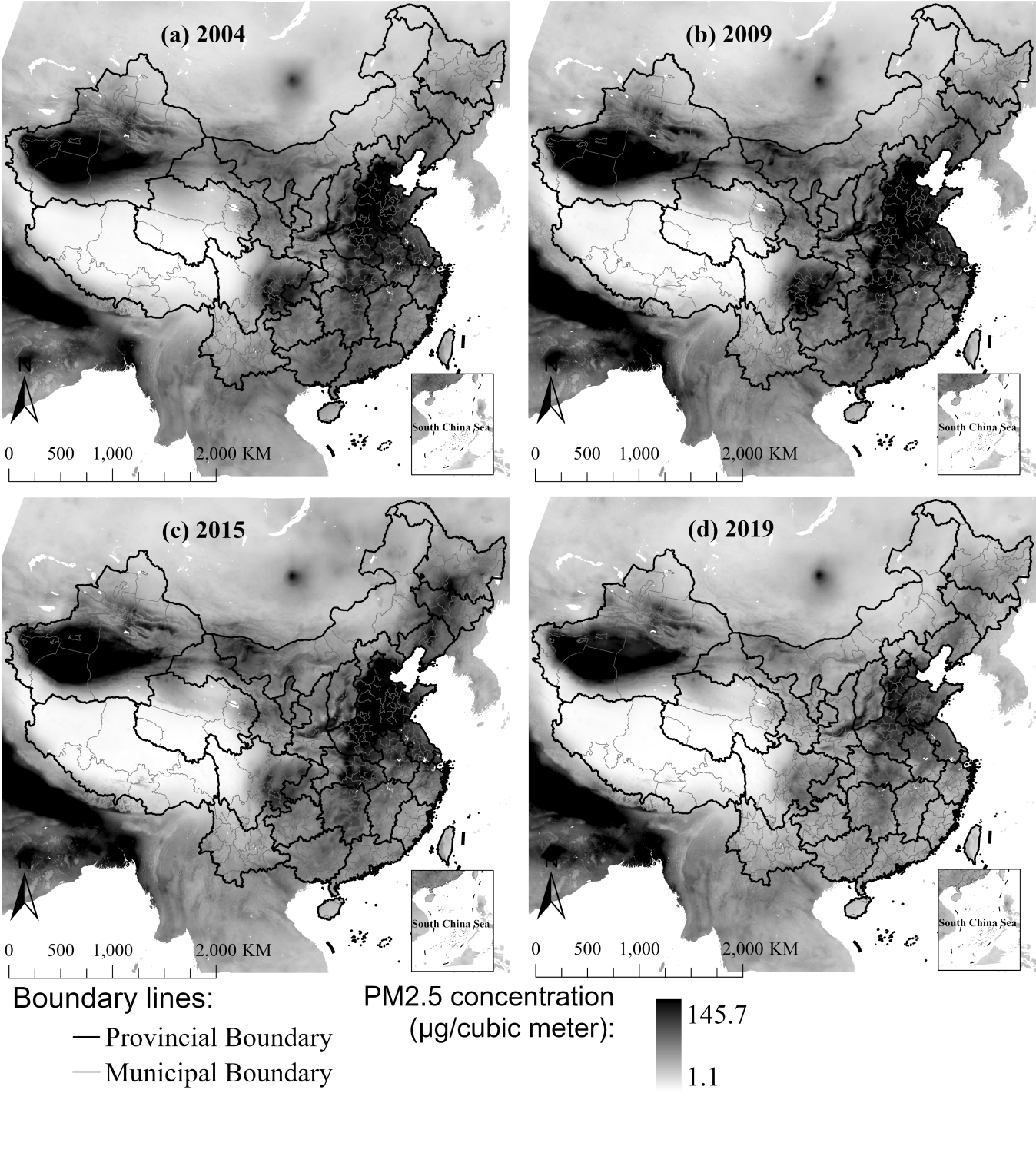

Fig. A.1 Raster data of PM2.5 concentration (van Donkelaar et al., 2021; sample of typical years)
Note: The basic map resources come from the National Geomatics Center of China (NGCC) and the Institute of Geographic Sciences and Natural Resources Research (IGSNRR), CAS.

**A2. Methods and Results of Spatial Autocorrelation Tests**

The implementation of spatial econometric analysis must be based on the existence of spatial autocorrelation of the explained variables, and its judging index is mainly Moran’s I of the global spatial autocorrelation test. The larger the absolute value of Moran’s I, the stronger the spatial autocorrelation. The Moran’s I is calculated as:

$Moran'sI=\frac{\sum_{i=1}^{N} \sum_{j=1}^{N} W_{ij}\left(Y_{i}-\bar{Y}\right)\left(Y_{j}-\bar{Y}\right)}{\left[\frac{1}{N}\sum_{j=1}^{N} \left(Y_{i}-\bar{Y}\right)^{2}\right]\sum_{i=1}^{N} \sum_{j=1}^{N} W_{ij}}$ (A.1)

The value range of Moran's I is [-1, 1]. In formula (A.1), *Y_i_* is the observed value of the variable in the i-th city, and $\bar{Y}$ is the mean value of all observed values. *W* represents the spatial weight matrix.

The local spatial autocorrelation analysis mainly focuses on the local spatial autocorrelation through Local Indicators of Spatial Association (LISA). The commonly used analysis methods of LISA are local Moran's I and Moran scatter plot. The local Moran's I of the i-th sample city can be calculated by the following formula:

$I_{i}=\frac{\left(Y_{i}-\bar{Y}\right)\sum_{j=1}^{N} W_{ij}\left(Y_{j}-\bar{Y}\right)}{\frac{1}{N}\sum_{i=1}^{N} \left(Y_{i}-\bar{Y}\right)^{2}}$ (A.2)

**Table A.1. Global Moran's I of PM2.5 concentration in 285 China’s cities, 2004–2019**

| Year | 2004 | 2005 | 2006 | 2007 | 2008 | 2009 | 2010 | 2011 | 2012 | 2013 | 2014 | 2015 | 2016 | 2017 | 2018 | 2019 |
| --- | --- | --- | --- | --- | --- | --- | --- | --- | --- | --- | --- | --- | --- | --- | --- | --- |
| I | 0.227 | 0.198 | 0.214 | 0.207 | 0.206 | 0.183 | 0.203 | 0.212 | 0.192 | 0.181 | 0.189 | 0.191 | 0.233 | 0.229 | 0.206 | 0.221 |
| Z(I) | 36.298 | 31.651 | 34.242 | 33.158 | 32.918 | 29.341 | 32.496 | 33.924 | 30.716 | 29.025 | 30.265 | 30.548 | 37.252 | 36.516 | 33.004 | 35.258 |
| P-value | 0.000 | 0.000 | 0.000 | 0.000 | 0.000 | 0.000 | 0.000 | 0.000 | 0.000 | 0.000 | 0.000 | 0.000 | 0.000 | 0.000 | 0.000 | 0.000 |

**a. 2004 b. 2012 c. 2019**


**Fig. A.2. Moran scatter plots of PM2.5 concentration in 285 China’s cities (three typical years).**Note: The horizontal axis is the value of the standardized Z statistics, and the vertical axis value is obtained by multiplying the spatial weight matrix W by the vector of Z statistics.

**A3. Complete estimation results of Spatial-GDID models**

Table A.2. Estimation results of Spatial-GDID models

|  | **Model A** | **Model B** | **Model C** | **Model D** | **Model E** | **Model F** | **Model G** |
| --- | --- | --- | --- | --- | --- | --- | --- |
| **Variables** | **Base-line Model** | **PSM-Spatial-GDID** | **Spatial-GDID-2SLS Model** | **Spatial weight matrix: *We*** | **Heterogeneity analysis: Eastern cities** | **Heterogeneity analysis: Administrative Level** | **Heterogeneity analysis: Pilot Cities of Emissions Trading** |
| *POST×FSDIOX* | -0.039*** | -0.047*** | -0.02** | -0.024*** | -0.049*** | -0.02** | -0.032*** |
|  | (-6.451) | (-8.33) | (-2.33) | (-4.786) | (-4.661) | (-2.09) | (-4.294) |
| *POST×FSDIOX×EAST* | - | - | - | - | 0.0143 | - | - |
|  | - | - | - | - | (1.159) | - | - |
| *POST×FSDIOX×CLEVEL* | - | - | - | - | - | -0.014** | - |
|  | - | - | - | - | - | (-2.537) | - |
| *POST×FSDIOX×PET* | - | - | - | - | - | - | -0.014* |
|  | - | - | - | - | - | - | (-1.739) |
| *GDPPC* | 0.145*** | 0.138*** | 0.132*** | 0.243*** | 0.143*** | 0.144*** | 0.151*** |
|  | (3.407) | (3.064) | (3.81) | (4.996) | (3.345) | (3.389) | (3.538) |
| *GDPPC^2^* | -0.007*** | -0.006*** | -0.006*** | -0.012*** | -0.006*** | -0.006*** | -0.007*** |
|  | (-2.913) | (-2.623) | (-3.36) | (-4.788) | (-2.844) | (-2.901) | (-3.015) |
| *POPUD* | -0.000509 | 0.001 | -0.001 | 0.005** | -0.001 | 0 | -0.001 |
|  | (-0.286) | (0.625) | (-0.86) | (2.525) | (-0.29) | (-0.275) | (-0.324) |
| *RDEMP* | -0.000099 | 0.004 | 0.001 | 0.002 | 0 | 0 | 0 |
|  | (-0.03) | (1.138) | (0.37) | (0.383) | (-0.04) | (0.012) | (0.145) |
| *ROAD* | -0.007 | -0.001 | -0.004 | -0.011** | -0.007 | -0.007* | -0.007* |
|  | (-1.618) | (-0.244) | (-1.11) | (-2.247) | (-1.637) | (-1.714) | (-1.682) |
| *GASR* | -0.011** | -0.009* | -0.01** | -0.021*** | -0.01** | -0.011** | -0.011** |
|  | (-2.045) | (-1.677) | (-2.35) | (-3.379) | (-2.023) | (-2.133) | (-2.052) |
| *NDVI* | -0.048 | -0.03 | -0.077** | -0.151*** | -0.045 | -0.05 | -0.045 |
|  | (-1.546) | (-0.923) | (-2.55) | (-5.153) | (-1.455) | (-1.625) | (-1.459) |
| *PRSD* | -1.759545 | 0.309 | -0.296 | 5.59*** | -1.821 | -1.659 | -1.711 |
|  | (-1.056) | (0.176) | (-0.2) | (3.335) | (-1.093) | (-0.997) | (-1.028) |
| *TEMP* | -14.067*** | -9.76*** | -10.759*** | 4.741*** | -14.074*** | -13.888*** | -14.035*** |
|  | (-8.213) | (-5.583) | (-5.21) | (4.536) | (-8.219) | (-8.118) | (-8.205) |
| *WIN* | -0.138*** | -0.113*** | -0.119*** | -0.146*** | -0.137*** | -0.14*** | -0.136*** |
|  | (-5.304) | (-4.345) | (-5.32) | (-6.62) | (-5.252) | (-5.389) | (-5.22) |
| *RHU* | -0.108 | 0.011 | -0.132** | 0.022 | -0.106 | -0.095 | -0.107 |
|  | (-1.599) | (0.158) | (-1.98) | (0.366) | (-1.57) | (-1.402) | (-1.587) |
| *W*×*POST×FSDIOX* | 0.119*** | 0.148*** | 0.022 | 0.216*** | 0.184*** | 0.34*** | 0.15*** |
|  | (4.225) | (5.648) | (0.64) | (3.063) | (3.295) | (3.164) | (3.594) |
| *W*×*POST×FSDIOX×EAST* | - | - | - | - | -0.085 | - | - |
|  | - | - | - | - | (-1.347) | - | - |
| *W*×*POST×FSDIOX×CLEVEL* | - | - | - | - | - | -0.192** | - |
|  | - | - | - | - | - | (-2.234) | - |
| *W*×*POST×FSDIOX×PET* | - | - | - | - | - | - | -0.084 |
|  | - | - | - | - | - | - | (-1.259) |
| *W*×*GDPPC* | 1.756*** | 0.964*** | 1.164*** | -1.772*** | 1.781*** | 1.732*** | 1.884*** |
|  | (5.362) | (2.906) | (4.71) | (-3.019) | (5.429) | (5.292) | (5.707) |
| *W*×*GDPPC^2^* | -0.09*** | -0.054*** | -0.059*** | 0.137*** | -0.092*** | -0.09*** | -0.096*** |
|  | (-5.403) | (-3.21) | (-4.65) | (4.263) | (-5.492) | (-5.423) | (-5.709) |
| *W*×*POPUD* | 0.098*** | 0.022 | 0.038*** | 0.023 | 0.097*** | 0.099*** | 0.096*** |
|  | (6.708) | (1.389) | (2.99) | (0.652) | (6.684) | (6.786) | (6.609) |
| *W*×*RDEMP* | -0.121*** | 0.022 | -0.013 | 0.746*** | -0.115*** | -0.115*** | -0.1*** |
|  | (-3.812) | (0.651) | (-0.48) | (9.943) | (-3.483) | (-3.61) | (-3.031) |
| *W*×*ROAD* | -0.136*** | 0.049 | -0.103*** | -0.749*** | -0.145*** | -0.131** | -0.153*** |
|  | (-2.607) | (0.939) | (-2.63) | (-9.387) | (-2.752) | (-2.5) | (-2.896) |
| *W*×*GASR* | -0.227*** | -0.136* | -0.102* | 0.132** | -0.222*** | -0.228*** | -0.239*** |
|  | (-3.379) | (-1.944) | (-1.89) | (2.24) | (-3.307) | (-3.397) | (-3.561) |
| *W*×*NDVI* | -0.742*** | -0.754*** | -0.065 | -0.125 | -0.762*** | -0.756*** | -0.776*** |
|  | (-4.412) | (-4.027) | (-0.39) | (-0.424) | (-4.513) | (-4.503) | (-4.6) |
| *W*×*PRSD* | 37.489*** | -2.94 | 12.746 | -271.275*** | 38.759*** | 37.439*** | 40.869*** |
|  | (3.763) | (-0.274) | (1.42) | (-9.946) | (3.868) | (3.762) | (4.066) |
| *W*×*TEMP* | 55.817*** | 29.411*** | 35.514*** | 0.512 | 56.297*** | 55.671*** | 56.413*** |
|  | (9.789) | (5.042) | (5.91) | (0.038) | (9.83) | (9.781) | (9.889) |
| *W*×*WIN* | -0.105 | -0.277** | -0.02 | -1.057*** | -0.112 | -0.116 | -0.118 |
|  | (-0.838) | (-2.262) | (-0.2) | (-2.828) | (-0.895) | (-0.928) | (-0.943) |
| *W*×*RHU* | 1.528*** | -0.767** | 1.329*** | 5.566*** | 1.51*** | 1.521*** | 1.509*** |
|  | (4.783) | (-2.191) | (4.56) | (8.006) | (4.723) | (4.77) | (4.721) |
| *W*×*PM* | 0.981*** | 0.977*** | 2.09*** | -0.075 | 0.981*** | 0.984*** | 0.983*** |
| (Interpreted Variable) | (304.057) | (244.672) | (19.54) | (-0.797) | (304.331) | (361.466) | (340.95) |
|  |  |  |  |  |  |  |  |
| *D.E._POST×FSDIOX* | -0.024*** | -0.030*** | -0.019** | -0.024*** | -0.022** | 0.062** | -0.005 |
|  | (-4.024) | (-6.091) | (-2.257) | (-4.882) | (-1.990) | (2.387) | (-0.629) |
| *I.E._ POST×FSDIOX* | 4.505*** | 4.449*** | 0.029** | 0.204*** | 8.496*** | 22.932*** | 7.534*** |
|  | (2.899) | (3.731) | (2.093) | (2.917) | (3.292) | (3.238) | (3.399) |
|  |  |  |  |  |  |  |  |
| Observations | 4560 | 3936 | 4560 | 4560 | 4560 | 4560 | 4560 |
| Number of cities | 285 | 285 | 285 | 285 | 285 | 285 | 285 |
| *R^2^* | 0.9736 | 0.9768 | 0.9736 | 0.9597 | 0.9736 | 0.9737 | 0.9736 |
| Log likelihood | 6735.4123 | 6107.02 | 6735.4123 | 5846.1324 | 6736.4185 | 6741.3881 | 6740.7272 |
| Wald spatial lag test | 401.585*** | 193.251*** | 100.208*** | 555.096*** | 400.756*** | 401.969*** | 404.417*** |
| LR spatial lag test | 407.764*** | 201.067*** | 407.764*** | 561.502*** | 406.938*** | 409.178*** | 411.546*** |
| Wald spatial error test | 451.871*** | 224.211*** | 109.747*** | 558.045*** | 451.17*** | 455.437*** | 456.579*** |
| LR spatial error test | 455.728*** | 232.09*** | 455.728*** | 559.485*** | 454.801*** | 459.925*** | 460.666*** |
| LR spatial fixed effects test | 11108.394 *** | 10086.787 *** | 10088.703 *** | 11108.394 *** | 11105.64 *** | 11116.038 *** | 11111.6 *** |
| LR time fixed effects test | 3186.68 *** | 3242.986 *** | 1578.417 *** | 3186.68 *** | 3189.912 *** | 3194.548 *** | 3173.147 *** |

Note: The t-statistics are marked in parentheses. *, **, and *** denote significance at 10%, 5%, and 1% levels, respectively. **D.E. before the variable symbol indicates direct effect, and I.E. indicates indirect effect.**

**A4. Effectiveness test results of Spatial-GDID model**

Table A.3. Mean standardized bias of variables before and after PSM.

| Year  Bias (%) | 2004 | 2005 | 2006 | 2007 | 2008 | 2009 | 2010 | 2011 | 2012 | 2013 | 2014 | 2015 | 2016 | 2017 | 2018 | 2019 |
| --- | --- | --- | --- | --- | --- | --- | --- | --- | --- | --- | --- | --- | --- | --- | --- | --- |
| Unmatched | 27.9 | 27.3 | 28.5 | 28.7 | 28.4 | 26.1 | 25.3 | 27.4 | 28.3 | 26.5 | 27.9 | 27.7 | 27.7 | 27.4 | 25.5 | 27.2 |
| Matched | 6.9 | 5.6 | 15.3 | 9.6 | 7.6 | 6.7 | 9.6 | 7.9 | 5.3 | 14.3 | 7.8 | 7.7 | 8.7 | 5.3 | 16.5 | 7.5 |

Note: Limited to space, only the mean standardization bias of all control variables are reported here. The specific matching results (balance test results) of typical years (2004, 2012, and 2019) are reported in Table A.3 of the supplementary materials.

Table A.4. Mean standardized bias of variables before and after PSM.

|  |  |  |  |  | 2004 |  |  |  |  |  |  | 2012 |  |  |  |  |  |  | 2019 |  |  |  |
| --- | --- | --- | --- | --- | --- | --- | --- | --- | --- | --- | --- | --- | --- | --- | --- | --- | --- | --- | --- | --- | --- | --- |
|  | Unmatched (U) |  | Mean |  |  | %reduct | t-test |  |  | Mean |  |  | %reduct | t-test |  |  | Mean |  |  | %reduct | t-test |  |
|  | Matched (M) |  | Treated | Untreated | Bias(%) | \|Bias\| | t | p>\|t\| |  | Treated | Untreated | Bias(%) | \|Bias\| | t | p>\|t\| |  | Treated | Untreated | Bias(%) | \|Bias\| | t | p>\|t\| |
| ***GDPPC*** | U |  | 8.7499 | 8.6422 | 17.5 |  | 1.34 | 0.181 |  | 9.6774 | 9.5895 | 15.1 |  | 1.14 | 0.254 |  | 10.046 | 9.9273 | 23.5 |  | 1.81 | 0.072 |
|  | M |  | 8.6668 | 8.6903 | -3.8 | 78.2 | -0.2 | 0.842 |  | 9.6805 | 9.613 | 11.6 | 23.2 | 0.66 | 0.513 |  | 10.109 | 10.033 | 15 | 36.1 | 0.8 | 0.427 |
|  |  |  |  |  |  |  |  |  |  |  |  |  |  |  |  |  |  |  |  |  |  |  |
| ***GDPPC^2^*** | U |  | 76.916 | 75.079 | 17.1 |  | 1.29 | 0.196 |  | 93.988 | 92.293 | 15.1 |  | 1.13 | 0.258 |  | 101.16 | 98.824 | 23 |  | 1.76 | 0.08 |
|  | M |  | 75.505 | 75.977 | -4.4 | 74.3 | -0.23 | 0.819 |  | 94.034 | 92.755 | 11.4 | 24.5 | 0.64 | 0.521 |  | 102.48 | 100.93 | 15.2 | 33.8 | 0.8 | 0.424 |
|  |  |  |  |  |  |  |  |  |  |  |  |  |  |  |  |  |  |  |  |  |  |  |
| ***POPUD*** | U |  | 7.2807 | 7.1459 | 11.8 |  | 0.91 | 0.364 |  | 7.9327 | 8.2237 | -39.9 |  | -3 | 0.003 |  | 7.9911 | 8.1626 | -26.5 |  | -2.01 | 0.046 |
|  | M |  | 7.075 | 7.1242 | -4.3 | 63.5 | -0.24 | 0.81 |  | 8.071 | 8.1407 | -9.6 | 76.1 | -0.53 | 0.594 |  | 8.0957 | 8.0846 | 1.7 | 93.5 | 0.09 | 0.924 |
|  |  |  |  |  |  |  |  |  |  |  |  |  |  |  |  |  |  |  |  |  |  |  |
| ***RDEMP*** | U |  | -7.1149 | -6.8277 | -34.8 |  | -2.59 | 0.01 |  | -6.7716 | -6.6208 | -17.9 |  | -1.35 | 0.179 |  | -6.609 | -6.5751 | -4.1 | -4.1 | -0.3 | 0.764 |
|  | M |  | -6.8822 | -6.8678 | -1.7 | 95 | -0.1 | 0.922 |  | -6.6263 | -6.6535 | 3.2 | 82 | 0.19 | 0.851 |  | -6.4456 | -6.5029 | 6.8 | -68.8 | 0.38 | 0.708 |
|  |  |  |  |  |  |  |  |  |  |  |  |  |  |  |  |  |  |  |  |  |  |  |
| ***ROAD*** | U |  | 9.6368 | 9.6148 | 2.5 |  | 0.19 | 0.851 |  | 10.264 | 10.192 | 9.4 |  | 0.71 | 0.477 |  | 10.706 | 10.593 | 15.4 |  | 1.17 | 0.243 |
|  | M |  | 9.635 | 9.5732 | 7.1 | -181.5 | 0.37 | 0.71 |  | 10.263 | 10.21 | 6.9 | 26.2 | 0.39 | 0.694 |  | 10.639 | 10.683 | -6 | 60.7 | -0.26 | 0.794 |
|  |  |  |  |  |  |  |  |  |  |  |  |  |  |  |  |  |  |  |  |  |  |  |
| ***GASR*** | U |  | -2.1132 | -2.1504 | 4.3 |  | 0.32 | 0.752 |  | -1.7447 | -1.7727 | 3.8 |  | 0.29 | 0.774 |  | -1.4897 | -1.5262 | 5.6 |  | 0.42 | 0.677 |
|  | M |  | -2.1953 | -2.1671 | -3.2 | 24.2 | -0.18 | 0.86 |  | -1.7456 | -1.7735 | 3.8 | 0.1 | 0.21 | 0.83 |  | -1.4702 | -1.5002 | 4.6 | 17.7 | 0.26 | 0.797 |
|  |  |  |  |  |  |  |  |  |  |  |  |  |  |  |  |  |  |  |  |  |  |  |
| ***NDVI*** | U |  | -0.3407 | -0.46035 | 36.4 |  | 3.22 | 0.001 |  | -0.3187 | -0.3860 | 24.8 |  | 2.15 | 0.032 |  | -0.3124 | -0.3610 | 17.1 |  | 1.45 | 0.149 |
|  | M |  | -0.4071 | -0.43201 | 7.6 | 79.2 | 0.4 | 0.692 |  | -0.3619 | -0.3647 | 1.1 | 95.7 | 0.06 | 0.951 |  | -0.38486 | -0.3671 | -6.3 | 63.4 | -0.33 | 0.744 |
|  |  |  |  |  |  |  |  |  |  |  |  |  |  |  |  |  |  |  |  |  |  |  |
| ***PRSD*** | U |  | 6.8736 | 6.8359 | 51.6 |  | 4.22 | 0 |  | 6.8722 | 6.8348 | 50.9 |  | 4.17 | 0 |  | 6.8721 | 6.8347 | 50.8 |  | 4.15 | 0 |
|  | M |  | 6.8347 | 6.8481 | -18.3 | 64.5 | -0.96 | 0.34 |  | 6.8437 | 6.8416 | 2.8 | 94.5 | 0.15 | 0.88 |  | 6.8513 | 6.845 | 8.5 | 83.2 | 0.5 | 0.619 |
|  |  |  |  |  |  |  |  |  |  |  |  |  |  |  |  |  |  |  |  |  |  |  |
| ***TEMP*** | U |  | 5.6632 | 5.65 | 73.2 |  | 5.91 | 0 |  | 5.6605 | 5.6464 | 75.1 |  | 5.99 | 0 |  | 5.6654 | 5.651 | 80.2 |  | 6.44 | 0 |
|  | M |  | 5.6564 | 5.6563 | 0.7 | 99 | 0.04 | 0.965 |  | 5.6507 | 5.6511 | -2.1 | 97.2 | -0.11 | 0.913 |  | 5.6595 | 5.6579 | 8.8 | 89 | 0.54 | 0.593 |
|  |  |  |  |  |  |  |  |  |  |  |  |  |  |  |  |  |  |  |  |  |  |  |
| ***WIN*** | U |  | 0.7275 | 0.7780 | -21.8 |  | -1.67 | 0.095 |  | 0.6860 | 0.7434 | -27.9 |  | -2.04 | 0.043 |  | 0.7482 | 0.8034 | -30.8 |  | -2.49 | 0.013 |
|  | M |  | 0.6970 | 0.726 | -12.6 | 42.3 | -0.72 | 0.472 |  | 0.7190 | 0.7222 | -1.6 | 94.4 | -0.09 | 0.929 |  | 0.7513 | 0.7452 | 3.4 | 89 | 0.2 | 0.842 |
|  |  |  |  |  |  |  |  |  |  |  |  |  |  |  |  |  |  |  |  |  |  |  |
| ***RHU*** | U |  | 4.1748 | 4.1065 | 36 |  | 3.03 | 0.003 |  | 4.1883 | 4.134 | 31 |  | 2.57 | 0.011 |  | 4.1849 | 4.145 | 22.3 |  | 1.79 | 0.074 |
|  | M |  | 4.1522 | 4.1298 | 11.8 | 67.2 | 0.6 | 0.547 |  | 4.1673 | 4.1608 | 3.7 | 88 | 0.21 | 0.836 |  | 4.1628 | 4.1528 | 5.5 | 75.1 | 0.28 | 0.783 |

Table A.5. Results using instrumental variables: Spatial-GDID-2SLS model.

|  | **Model A** | **Model H** |
| --- | --- | --- |
| **Variables** | **Base-line Spatial-GDID Model** | **Spatial-GDID-2SLS Model** |
| *POST×FSDIOX* | -0.039*** | -0.02** |
|  | (-6.451) | (-2.33) |
| *GDPPC* | 0.145*** | 0.132*** |
|  | (3.407) | (3.81) |
| *GDPPC^2^* | -0.007*** | -0.006*** |
|  | (-2.913) | (-3.36) |
| *POPUD* | -0.001 | -0.001 |
|  | (-0.286) | (-0.86) |
| *RDEMP* | -0.0001 | 0.001 |
|  | (-0.03) | (0.37) |
| *ROAD* | -0.007 | -0.004 |
|  | (-1.618) | (-1.11) |
| *GASR* | -0.011** | -0.01** |
|  | (-2.045) | (-2.35) |
| *NDVI* | -0.048 | -0.077** |
|  | (-1.546) | (-2.55) |
| *PRSD* | -1.760 | -0.296 |
|  | (-1.056) | (-0.2) |
| *TEMP* | -14.067*** | -10.759*** |
|  | (-8.213) | (-5.21) |
| *WIN* | -0.138*** | -0.119*** |
|  | (-5.304) | (-5.32) |
| *RHU* | -0.108 | -0.132** |
|  | (-1.599) | (-1.98) |
| *W*×*POST×FSDIOX* | 0.120*** | 0.022 |
|  | (4.225) | (0.64) |
| *W*×*GDPPC* | 1.756*** | 1.164*** |
|  | (5.362) | (4.71) |
| *W*×*GDPPC^2^* | -0.09*** | -0.059*** |
|  | (-5.403) | (-4.65) |
| *W*×*POPUD* | 0.098*** | 0.038*** |
|  | (6.708) | (2.99) |
| *W*×*RDEMP* | -0.121*** | -0.013 |
|  | (-3.812) | (-0.48) |
| *W*×*ROAD* | -0.136*** | -0.103*** |
|  | (-2.607) | (-2.63) |
| *W*×*GASR* | -0.227*** | -0.102* |
|  | (-3.379) | (-1.89) |
| *W*×*NDVI* | -0.742*** | -0.065 |
|  | (-4.412) | (-0.39) |
| *W*×*PRSD* | 37.489*** | 12.746 |
|  | (3.763) | (1.42) |
| *W*×*TEMP* | 55.817*** | 35.514*** |
|  | (9.789) | (5.91) |
| *W*×*WIN* | -0.105 | -0.02 |
|  | (-0.838) | (-0.2) |
| *W*×*RHU* | 1.528*** | 1.329*** |
|  | (4.783) | (4.56) |
| *W*×*PM* | 0.981*** | 2.09*** |
| (Interpreted Variable) | (304.057) | (19.54) |
| *D.E._POST×FSDIOX* | -0.024*** | -0.019** |
|  | (-4.024) | (-2.257) |
| *I.E._ POST×FSDIOX* | 4.505*** | 0.029** |
|  | (2.899) | (2.093) |
|  |  |  |
| Observations | 4560 | 4560 |
| Number of cities | 285 | 285 |
| *R^2^* | 0.974 | 0.896 |
| Log likelihood | 6735.412 | - |
| Underidentification test  (Kleibergen-Paap rk LM statistic) | - | 314.405*** |
| Weak identification test  (Kleibergen-Paap rk Wald F statistic) | - | 65.456 |
| Overidentification test of all instruments  (Hansen J statistic): | - | 2.048  (P-value=0.727) |

Note: The t-statistics are marked in parentheses. *, **, and *** denote significance at 10%, 5%, and 1% levels, respectively. **D.E. before the variable symbol indicates direct effect, and I.E. indicates indirect effect.** The results in this table are estimated by stata17 and the script provided by Schaffer (2010).

**A5. Mediation effect analysis**

**Table A.6. Mediation effect analysis results.**

|  | **Model A** | **Mediation effects: SO_2_ emission intensity** |  | **Mediation effects: Industrial employment** |  | **Mediation effects: Industrial fixed assets** |  |
| --- | --- | --- | --- | --- | --- | --- | --- |
| **Variables** | **Base-line Spatial-GDID Model** | **Stage 1** | **Stage 2** | **Stage 1** | **Stage 2** | **Stage 1** | **Stage 2** |
| *POST×FSDIOX* | -0.039*** | -0.216*** | -0.032*** | -0.049*** | -0.038*** | -0.119*** | -0.038*** |
|  | (-6.451) | (-7.424) | (-3.767) | (-3.31) | (-6.378) | (-3.527) | (-6.316) |
| *SDIOXINT* | - | - | -0.007 | - | - | - | - |
|  | - | - | (-1.354) | - | - | - | - |
| *NDEMP* | - | - | - | - | 0.000003 | - | - |
|  | - | - | - | - | (0.455) | - | - |
| *NDFA* | - | - | - | - | - | - | 0.007** |
|  | - | - | - | - | - | - | (2.507) |
| *GDPPC* | 0.145*** | -2.459*** | 0.106** | 1.165*** | 0.145*** | 4.127*** | 0.142*** |
|  | (3.407) | (-15.842) | (2.248) | (11.063) | (3.412) | (17.302) | (3.231) |
| *GDPPC^2^* | -0.007*** | 0.073*** | -0.005** | -0.051*** | -0.007*** | -0.181*** | -0.007*** |
|  | (-2.913) | (8.94) | (-2.138) | (-9.247) | (-2.921) | (-14.483) | (-2.876) |
| *POPUD* | -0.000509 | 0.007 | -0.001 | -0.007 | -0.001 | -0.014 | 0 |
|  | (-0.286) | (1.07) | (-0.486) | (-1.637) | (-0.345) | (-1.377) | (-0.04) |
| *RDEMP* | -0.000099 | -0.063*** | -0.005 | -0.018** | 0 | -0.025 | -0.001 |
|  | (-0.03) | (-5.017) | (-1.306) | (-2.171) | (-0.05) | (-1.362) | (-0.31) |
| *ROAD* | -0.007 | 0.026* | -0.006 | 0.004 | -0.007* | -0.01 | -0.007* |
|  | (-1.618) | (1.696) | (-1.33) | (0.423) | (-1.677) | (-0.436) | (-1.741) |
| *GASR* | -0.011** | 0.012 | -0.007 | 0.011 | -0.01** | -0.024 | -0.011** |
|  | (-2.045) | (0.69) | (-1.279) | (0.851) | (-2.017) | (-0.837) | (-2.085) |
| *NDVI* | -0.048 | -0.049 | -0.056* | 0.163** | -0.049 | -0.27 | -0.056* |
|  | (-1.546) | (-0.439) | (-1.74) | (2.124) | (-1.58) | (-1.55) | (-1.825) |
| *PRSD* | -1.760 | -21.406*** | 2.13 | -3.956 | -1.667 | 37.719*** | -2.111 |
|  | (-1.056) | (-3.094) | (1.052) | (-0.96) | (-1.001) | (4.044) | (-1.272) |
| *TEMP* | -14.067*** | 0.565 | -10.493*** | -8.703** | -14.036*** | 8.982 | -14.066*** |
|  | (-8.213) | (0.093) | (-5.918) | (-2.056) | (-8.204) | (0.937) | (-8.263) |
| *WIN* | -0.138*** | 0.187** | -0.128*** | -0.002 | -0.138*** | 0.086 | -0.144*** |
|  | (-5.304) | (1.962) | (-4.615) | (-0.033) | (-5.296) | (0.588) | (-5.575) |
| *RHU* | -0.108 | -0.271 | -0.01 | -0.542*** | -0.106 | 0.576 | -0.098 |
|  | (-1.599) | (-1.131) | (-0.15) | (-3.255) | (-1.568) | (1.528) | (-1.469) |
| *W*×*POST×FSDIOX* | 0.119*** | 0.709*** | 0.105*** | 0.144** | 0.116*** | 0.54*** | 0.106*** |
|  | (4.225) | (5.31) | (2.679) | (2.052) | (4.097) | (3.4) | (3.77) |
| *W*×*SDIOXINT* | - | - | 0.257*** | - | - | - | - |
|  | - | - | (9.063) | - | - | - | - |
| *W*×*INDEMP* | - | - | - | - | 0*** | - | - |
|  | - | - | - | - | (2.887) | - | - |
| *W*×*INDFA* | - | - | - | - | - | - | 0.19*** |
|  | - | - | - | - | - | - | (6.574) |
| *W*×*GDPPC* | 1.756*** | 10.956*** | 1.039*** | -2.771*** | 1.737*** | -0.648 | 0.717** |
|  | (5.362) | (9.409) | (3.02) | (-3.423) | (5.311) | (-0.345) | (1.981) |
| *W*×*GDPPC^2^* | -0.09*** | -0.541*** | -0.036** | 0.144*** | -0.089*** | -0.046 | -0.038** |
|  | (-5.403) | (-9.087) | (-2.028) | (3.495) | (-5.32) | (-0.482) | (-2.081) |
| *W*×*POPUD* | 0.098*** | 0.318*** | 0.076*** | -0.149*** | 0.097*** | -0.234*** | 0.111*** |
|  | (6.708) | (6.431) | (5.212) | (-4.123) | (6.675) | (-2.852) | (7.595) |
| *W*×*RDEMP* | -0.121*** | -1.1*** | -0.132*** | 0.258*** | -0.121*** | 0.122 | -0.073** |
|  | (-3.812) | (-8.444) | (-3.355) | (3.279) | (-3.816) | (0.683) | (-2.262) |
| *W*×*ROAD* | -0.136*** | -0.859*** | -0.109* | -0.167 | -0.147*** | 0.64** | -0.123** |
|  | (-2.607) | (-3.839) | (-1.667) | (-1.292) | (-2.814) | (2.185) | (-2.363) |
| *W*×*GASR* | -0.227*** | -0.102 | -0.098 | -0.027 | -0.214*** | -0.246 | -0.228*** |
|  | (-3.379) | (-0.426) | (-1.406) | (-0.165) | (-3.181) | (-0.656) | (-3.424) |
| *W*×*NDVI* | -0.742*** | -1.485** | -0.829*** | -1.234*** | -0.747*** | 3.689*** | -0.762*** |
|  | (-4.412) | (-2.518) | (-4.812) | (-2.967) | (-4.445) | (3.906) | (-4.549) |
| *W*×*PRSD* | 37.489*** | 236.228*** | 28.469** | -57.757** | 35.446*** | 96.929* | 25.252** |
|  | (3.763) | (5.812) | (2.324) | (-2.344) | (3.554) | (1.723) | (2.511) |
| *W*×*TEMP* | 55.817*** | 20.621 | 47.002*** | 11.975 | 54.854*** | -13.389 | 54.552*** |
|  | (9.789) | (1.038) | (8.085) | (0.85) | (9.616) | (-0.42) | (9.621) |
| *W*×*WIN* | -0.105 | 0.712 | -0.153 | 0.063 | -0.11 | 0.388 | -0.08 |
|  | (-0.838) | (1.58) | (-1.154) | (0.202) | (-0.878) | (0.553) | (-0.646) |
| *W*×*RHU* | 1.528*** | 6.826*** | 0.41 | -0.139 | 1.451*** | 0.686 | 1.264*** |
|  | (4.783) | (5.955) | (1.2) | (-0.176) | (4.531) | (0.383) | (3.95) |
| *W*×*PM* | 0.981*** | - | 0.981*** | - | 0.982*** | - | 0.983*** |
| (Interpreted Variable) | (304.057) | - | (296.573) | - | (321.912) | - | (342.02) |
| *W*×*SDIOXINT* | - | 0.966*** | - | - | - | - | - |
| (Interpreted Variable) | - | (176.215) | - | - | - | - | - |
| *W*×*INDEMP* | - | - | - | 0.764*** | - | - | - |
| (Interpreted Variable) | - | - | - | (20.184) | - | - | - |
| *W*×*INDFA* | - | - | - | - | - | 0.089 | - |
| (Interpreted Variable) | - | - | - | - | - | (0.873) | - |
|  |  |  |  |  |  |  |  |
| *D.E._POST×FSDIOX* | -0.024*** | -0.166*** | -0.015** | -0.051*** | -0.023*** | -0.122*** | -0.023*** |
|  | (-4.024) | (-6.367) | (-2.543) | (-3.644) | (-4.180) | (-3.960) | (-4.975) |
| *I.E._ POST×FSDIOX* | 4.505*** | 14.795*** | 4.303** | 0.541** | 4.069** | 0.591*** | 3.740*** |
|  | (2.899) | (3.618) | (2.296) | (2.084) | (2.409) | (3.846) | (3.405) |
|  |  |  |  |  |  |  |  |
| Observations | 4560 | 3990 | 3990 | 4560 | 4560 | 4560 | 4560 |
| Number of cities | 285 | 285 | 285 | 285 | 285 | 285 | 285 |
| *R^2^* | 0.9736 | 0.9775 | 0.9746 | 0.8776 | 0.9736 | 0.8227 | 0.9739 |
| Log likelihood | 6735.4123 | 1193.0747 | 6112.0647 | 2658.8894 | 6739.9732 | -1046.647 | 6761.9302 |
| Wald spatial lag test | 401.585*** | 435.449*** | 394.229*** | 101.053*** | 410.399*** | 82.209*** | 446.722*** |
| LR spatial lag test | 407.764*** | 440.388*** | 402.064*** | 106.399*** | 416.699*** | 87.31*** | 452.415*** |
| Wald spatial error test | 451.871*** | 486.241*** | 516.752*** | 93.575*** | 460.906*** | 74.272*** | 503.264*** |
| LR spatial error test | 455.728*** | 479.509*** | 516.573*** | 97.742*** | 464.796*** | 83.39*** | 505.216*** |
| LR spatial fixed effects test | 11108.394 *** | 7569.855 *** | 10152.776 *** | 7098.88 *** | 11100.33 *** | 4723.577 *** | 10955.857 *** |
| LR time fixed effects test | 3186.68 *** | 1349.698 *** | 1266.927 *** | 491.149 *** | 3185.683 *** | 467.598 *** | 3087.133 *** |

Note: The t-statistics are marked in parentheses. *, **, and *** denote significance at 10%, 5%, and 1% levels, respectively. **D.E. before the variable symbol indicates direct effect, and I.E. indicates indirect effect.**
